# Minimally invasive lymphocele drainage using the Da Vinci® single port platform: step-by- step technique

**DOI:** 10.1590/S1677-5538.IBJU.2021.0272

**Published:** 2021-08-30

**Authors:** Sunil Reddy, Marcio Covas Moschovas, Seetharam Bhat, Jonathan Noel, Talia Helman, Roshane Perera, Travis Rogers, Vipul Patel

**Affiliations:** 1 AdventHealth Global Robotics Institute Celebration FL USA AdventHealth Global Robotics Institute, Celebration, FL, USA; 2 University of Central Florida College of Medicine Orlando FL USA University of Central Florida College of Medicine, Orlando, FL, USA; 3 University of Florida Gainesville FL USA University of Florida, Gainesville, FL, USA

## Abstract

**Background:**

Reports in the literature describe lymphocele formation in up to half of patients following pelvic lymph node dissection (PLND) ( 1 ) in robotic-assisted radical prostatectomy (RARP), with 1-2% requiring intervention ( 2 ). The advantage of surgical approach is permanent excision of the lymphocele capsule and fewer days with pelvic drains compared to percutaneous drainage. This study aims to describe the step-by-step surgical management of symptomatic lymphoceles using a less invasive robotic platform, the Da Vinci® Single Port (SP).

**Material and Methods:**

We describe the technique of lymphocelectomy and marsupialization with the Da Vinci® SP for symptomatic lymphocele. For this study, several treatment modalities for symptomatic lymphoceles were available, including percutaneous drainage, sclerosing agents, and surgical marsupialization. All the data for this study were obtained through the procedure via Da Vinci® SP.

**Results:**

Operative time for the case was 84 minutes. Blood loss was 25ml. No intra- or post- operative complications were reported. The patient had his drain removed in under 24 hours after surgery. The mean follow-up period was 7.7 months. There were no complications or lymphocele recurrence.

**Conclusion:**

Da Vinci® SP lymphocelectomy is safe and feasible with satisfactory outcomes. The SP enables definitive treatment of the lymphocele sac ( 3 ), reducing the number of days with abdominal drains and allows further decrease in surgical invasiveness with fewer incisions and better cosmesis.

**Figure:** Available at:http://www.intbrazjurol.com.br/video-section/20210272_Reddy_et_al
